# Using Inflammatory Plasma Biomarkers to Predict Preclinical Alzheimer’s Disease

**DOI:** 10.1002/alz.091932

**Published:** 2025-01-09

**Authors:** Haley Leclerc, Jessica Alber, Zachary J. Kunicki, Sid E. O'Bryant, Athene KW Lee

**Affiliations:** ^1^ University of Rhode Island, Kingston, RI USA; ^2^ Alpert Medical School of Brown University, Providence, RI USA; ^3^ Butler Hospital Memory and Aging Program, Providence, RI USA; ^4^ George & Anne Ryan Institute for Neuroscience, Kingston, RI USA; ^5^ Lifespan ‐ Rhode Island Hospital, Providence, RI USA; ^6^ Butler Hospital, Providence, RI USA; ^7^ University of North Texas Health Science Center, Fort Worth, TX USA; ^8^ Warren Alpert Medical School of Brown University, Providence, RI USA

## Abstract

**Background:**

Plasma biomarkers have recently emerged to detect symptomatic Alzheimer Disease (AD), but have yet to be validated in preclinical AD populations, where Aβ accumulates in the brain but older adults are cognitively unimpaired (CU). In addition to AD pathologic plasma biomarkers (amyloid and tau), inflammatory markers can accurately detect symptomatic AD. We used pathologic and inflammatory plasma biomarkers to predict amyloid PET status in CU older adults.

**Method:**

Participants were 125 CU older adults (mean age = 68) from the Butler Hospital Alzheimer’s Prevention Registry who completed amyloid PET through a separate research study. Blood samples were collected and analyzed for the following: an inflammatory panel consisting of 21 proteins, Aβ40, Aβ42, tau (total), p‐tau181, p‐tau 231, p‐tau217, GFAP and NfL. Multiple regression was used to evaluate the best predictors of amyloid PET status (positive vs. negative) in CU older adults. Model 1 included predictors age, education, and gender. Model 2 and 3 added predictors APOE status, Aβ42/40 ratio and p‐tau181 respectively. Random forest (RF) modeling was used to establish the five proteomic markers that best predicted amyloid PET status, and these markers were added in Model 4.

**Result:**

We tested RF models that included all 21 inflammatory proteins, as well as the top 10, 8 and 5 inflammatory proteins. Models were compared based on sensitivity, specificity, PPV, NPV, and AUC. The model including the top 5 inflammatory proteins (CRP, SAA, sVCAM1, TNFα, and TARC) was the best fit with sensitivity of .88, specificity of .31, PPV of .71, NPV of .57 and AUC of .63. Multiple regression analysis showed that the best model for predicting amyloid PET positivity included age, years of education, gender, APOE E4 status, Aβ42/40 ratio and p‐tau181(p<.01). Adding the top 5 proteomic markers did not significantly improve the model.

**Conclusion:**

Results revealed that the proteomic inflammatory markers in plasma did not add predictive value to standard AD pathologic plasma biomarkers in predicting amyloid PET positivity in CU older adults. This may reflect that the changes associated with inflammatory biomarkers occur later downstream in the pathogenesis and disease progression of AD.

